# Comparing a Dedicated Breast Spectral CT Protocol With Standard MonoE40 Reconstruction for Breast Cancer Characterization and Opportunistic Assessment

**DOI:** 10.7759/cureus.117243

**Published:** 2026-09-30

**Authors:** Ashton Padowitz, Zane Sherif, Kirralee Sherif, Amanda Winspear

**Affiliations:** 1 Radiology, Mermaid Beach Radiology, Gold Coast, AUS

**Keywords:** breast cancer imaging, breast imaging, case report, computed tomography, iodine mapping, opportunistic screening, spectral ct

## Abstract

Breast malignancies can be difficult to assess on conventional imaging, particularly in patients with heterogeneous or dense breast tissue. We report the case of a woman in her early 70s with a biopsy-confirmed right breast malignancy who underwent contrast-enhanced spectral CT with both standard MonoE40 reconstruction and a dedicated breast spectral CT protocol. The dedicated reconstruction improved visualization of the primary lesion, associated dermal changes, ipsilateral axillary nodal abnormalities, and tumor vascularity, while also providing additional iodine-based enhancement information. These reconstructions were generated from the same CT acquisition and therefore required no additional radiation exposure or image acquisition. Subsequent surgical pathology confirmed locally advanced breast carcinoma with skin and regional nodal involvement, providing correlation with several of the imaging abnormalities. This case demonstrates the technical feasibility and qualitative added imaging information of dedicated breast spectral CT reconstruction in the assessment of a known breast malignancy undergoing preoperative staging.

## Introduction

Breast cancer remains a major cause of cancer-related morbidity and mortality among women worldwide. Mammography is the primary imaging modality used for breast cancer detection; however, its diagnostic sensitivity decreases in women with dense breast tissue [1]. Mammographic sensitivity has been reported to decrease from approximately 88% in predominantly fatty breasts to approximately 62% in women with extremely dense breast tissue [1]. Dense fibroglandular tissue may obscure malignancy on mammography and is itself an independent risk factor for breast cancer development [2].

Spectral CT provides additional tissue characterization information through techniques including iodine mapping and virtual monoenergetic reconstruction at 40 keV. In a prospective single-center, case-control study of 50 women with histopathologically confirmed unilateral breast cancer undergoing staging CT, Hasse et al. reported high diagnostic performance for MonoE40 spectral CT reconstruction, with a sensitivity of 96% and specificity of 94% for breast cancer detection [3]. Spectral CT may also improve lesion conspicuity and assessment of contrast enhancement, while iodine mapping may assist in differentiating enhancing malignant tissue from surrounding breast parenchyma [3].

Although CT is not used as a primary breast cancer screening modality because of radiation exposure considerations [4], thoracic CT examinations frequently include breast tissue within the acquired field. Breast abnormalities may therefore be identified incidentally when CT is performed for another clinical indication. In contrast, opportunistic breast assessment refers to the deliberate evaluation of breast tissue using data that has already been acquired as part of a clinically indicated CT examination. In this case, the malignancy was already biopsy-confirmed, and the dedicated spectral reconstruction was used for preoperative staging and further characterization rather than screening or incidental detection.

We present a case of biopsy-confirmed right breast malignancy assessed using both standard MonoE40 spectral CT reconstruction and a dedicated breast spectral CT protocol, with a qualitative within-patient comparison of lesion conspicuity, enhancement characteristics, vascular visualization, and axillary nodal assessment.

## Case presentation

A woman in her early 70s with a previously biopsy-confirmed right breast malignancy underwent a clinically indicated contrast-enhanced spectral CT of the chest for preoperative staging and further assessment. Core biopsy performed before this examination demonstrated extensive infiltration by invasive carcinoma arranged in linear cords and tubules, provisionally graded as grade 2. Immunohistochemistry (IHC) demonstrated estrogen receptor positivity (90%), weak progesterone receptor positivity (10%), and human epidermal growth factor receptor 2 (HER2) negativity (IHC 1+). E-cadherin was positive, suggestive of an invasive carcinoma. The standard MonoE40 and dedicated breast images were reconstructions generated from the same spectral CT acquisition rather than separate CT examinations. Her persistent clinical symptom was a painless right breast mass, with examination demonstrating a palpable retro-areolar mass and associated nipple inversion.

Contrast-enhanced spectral CT of the chest was performed in the prone position on a Philips IQon Spectral CT scanner using a single-phase, triple-bolus intravenous contrast technique. Acquisition was performed at 120 kV and 133 mAs. A total of 30 mL of iodinated contrast at a concentration of 300 mg/mL was administered. Injection rate and exact scan timing were not available from the retained study metadata. Images were reconstructed using both the standard thoracic MonoE40 reconstruction and an in-house dedicated breast spectral CT protocol incorporating enhancement mapping and three-dimensional breast reconstruction using Philips IntelliSpace Portal, software version 15.0.6.471. The dedicated breast reconstruction used a standard reconstruction kernel, spectral 6 filter, average render mode, and a 512 x 512 matrix, and display settings of W255/L128. MonoE40 was selected as the comparator because it represented the routinely used spectral reconstruction for thoracic CT at our institution. The standard MonoE40 reconstruction used a slice thickness of 0.67 mm, whereas the dedicated breast reconstruction used a slice thickness of 3.17 mm. The standard and dedicated reconstructions were compared qualitatively within the same patient by a single radiologist; no blinded reader study or formal quantitative analysis of lesion conspicuity was performed. Both reconstructions were generated from the same CT acquisition and therefore required no additional radiation exposure, contrast administration, or image acquisition. The total dose-length product was 330.4 mGy·cm. Estimated effective dose was calculated by multiplying the dose-length product by the adult chest conversion coefficient of 0.014 mSv/(mGy·cm), giving an estimated effective radiation dose of approximately 4.63 mSv.

The standard MonoE40 reconstruction demonstrated a spiculated retro-areolar right breast mass measuring approximately 28 × 32 × 19 mm, with central calcification, nipple inversion, and associated dermal thickening (Figures 1A, 1B). On qualitative single radiologist assessment, the dedicated breast spectral CT reconstruction appeared to provide greater conspicuity of the lesion’s irregular margins and enhancement characteristics compared with the standard MonoE40 reconstruction. Enhancement mapping also appeared to provide clearer differentiation between the enhancing lesion and adjacent breast parenchyma, as well as improved visualization of the associated nipple inversion and dermal thickening (Figures 2A, 2B).

**Figure 1:**
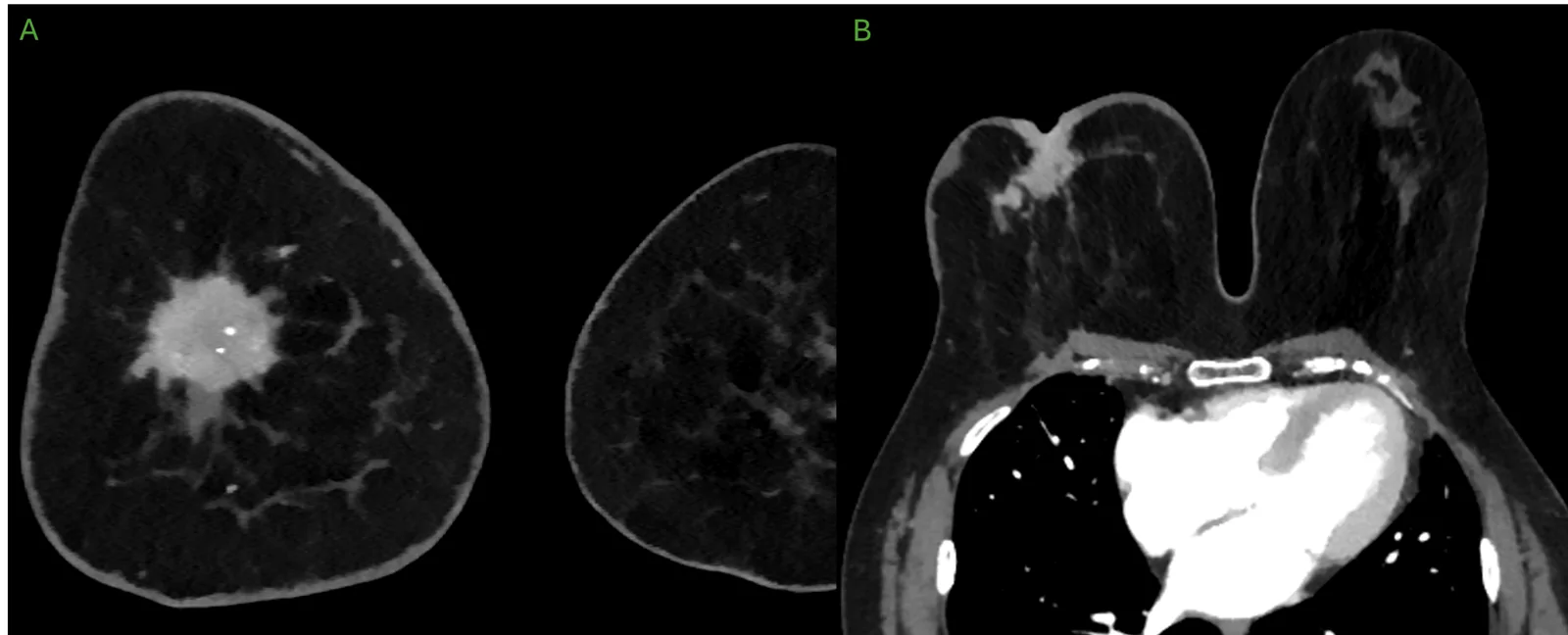
Standard MonoE40 spectral CT reconstructions demonstrating the spiculated right breast lesion. (A) Anterior coronal reconstruction demonstrating central calcification within the lesion. (B) Axial reconstruction demonstrating the retro-areolar mass, associated nipple inversion, and dermal thickening. Heterogeneous fibroglandular tissue is present in the left breast.

**Figure 2:**
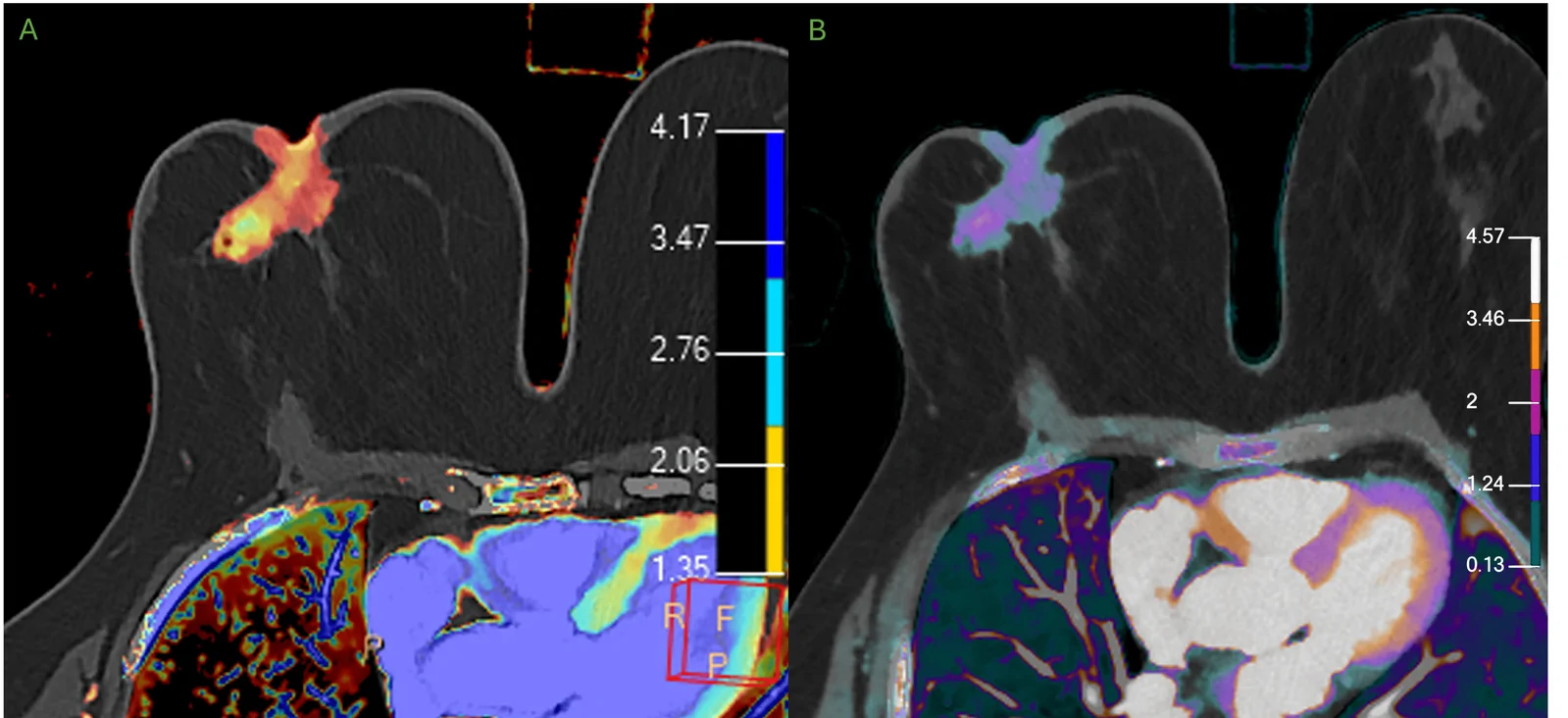
Dedicated breast spectral CT reconstructions demonstrating the spiculated right breast lesion. (A) Axial reconstruction using a narrow threshold range for enhancement mapping of 1.35 to 4.17, demonstrating the mass, nipple inversion, and dermal thickening. (B) Corresponding reconstruction using a wider enhancement threshold of 0.13 to 4.57. Heterogeneous fibroglandular tissue is present in the left breast.

Hyper-enhancing right axillary lymph nodes were identified, with the largest measuring approximately 9 × 13 mm. The nodes demonstrated rounded morphology and cortical thickening in addition to hyperenhancement, features considered suspicious for metastatic nodal involvement. On qualitative single radiologist assessment, these nodes appeared more conspicuous on the dedicated breast reconstruction than on the corresponding standard MonoE40 images (Figures 3A, 3B). Iodine density was measured using a region of interest (ROI) placed within the peripheral enhancing component of the tumor. The lesion ROI measured 23.71 mm² and had a mean iodine density of 2.30 mg/mL. A separate background ROI measuring 28.98 mm² was placed in adjacent non-enhancing breast tissue and demonstrated a mean iodine density of approximately -0.05 mg/mL (Figure 4). Three-dimensional reconstruction demonstrated continuity of a prominent enhancing arterial branch supplying the lesion with the right internal mammary artery (Figures 5A, 5B). No definite metastatic disease was identified within the anatomical field covered by the chest CT examination.

**Figure 3:**
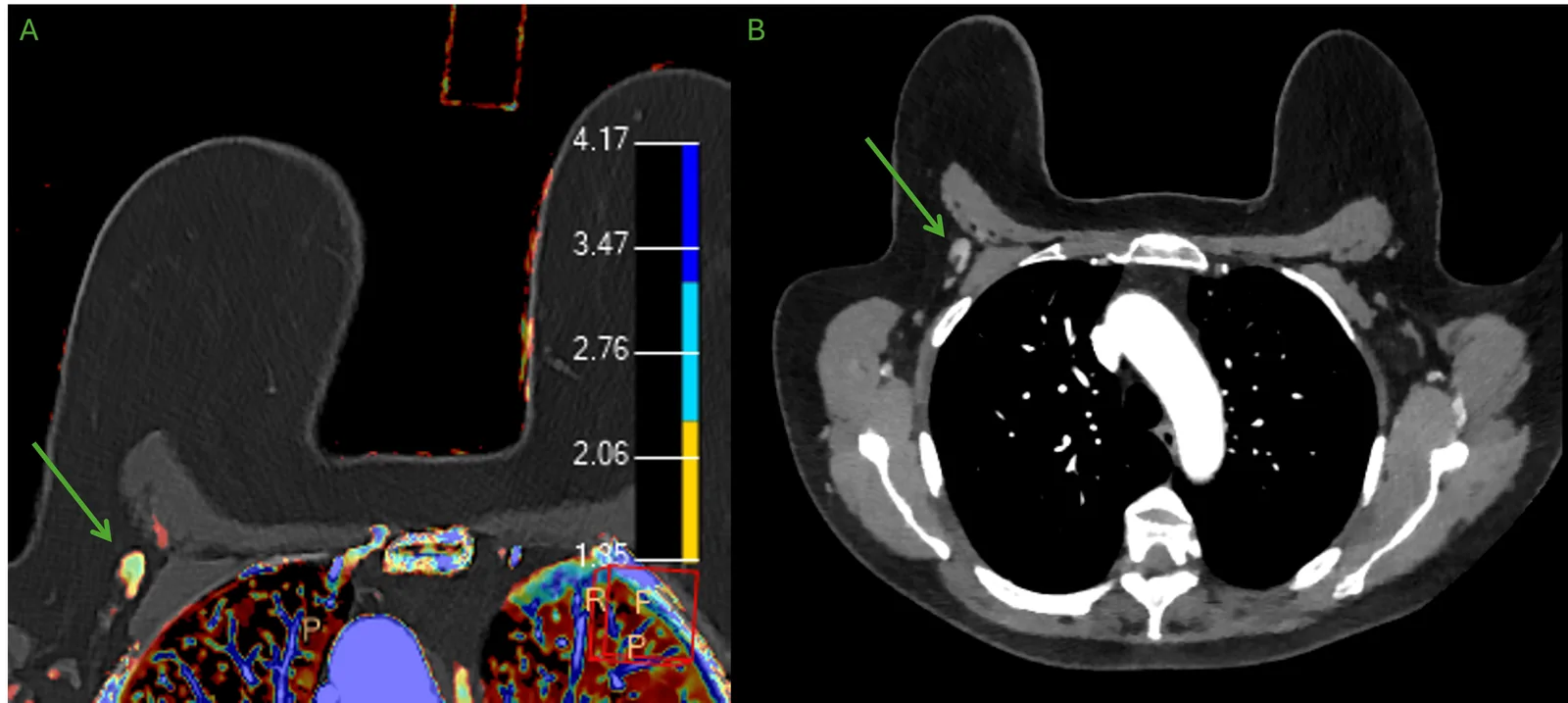
Comparison of the ipsilateral axillary lymph nodes at corresponding slice levels. (A) Dedicated breast spectral CT reconstruction demonstrating enhanced conspicuity of the right axillary lymph nodes. (B) Standard MonoE40 reconstruction. The suspicious lymph nodes are indicated by the green arrows.

**Figure 4:**
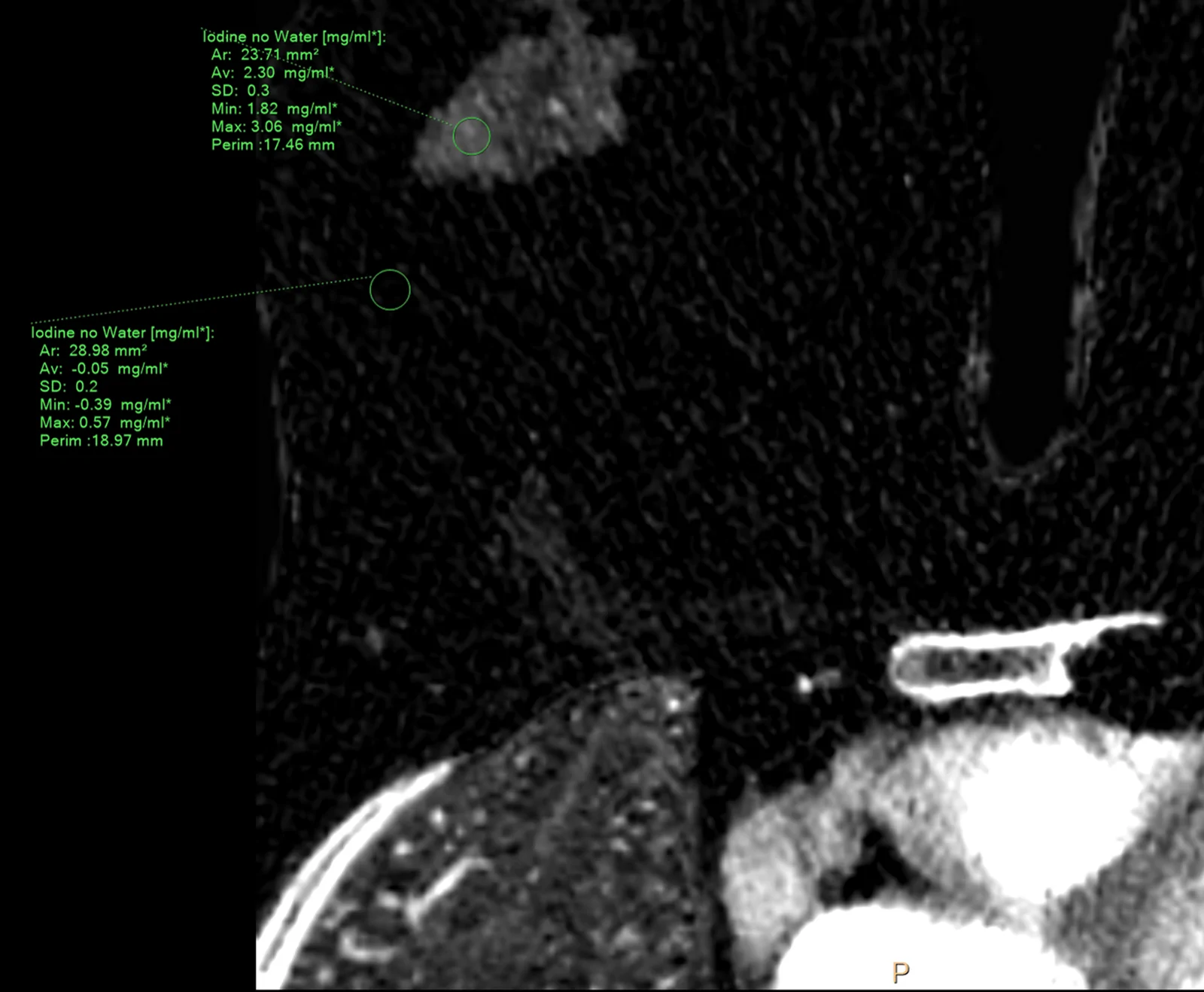
Iodine map demonstrating increased iodine concentration within the enhancing right breast lesion. The average peripheral iodine density was approximately 2.3 mg/mL.

**Figure 5:**
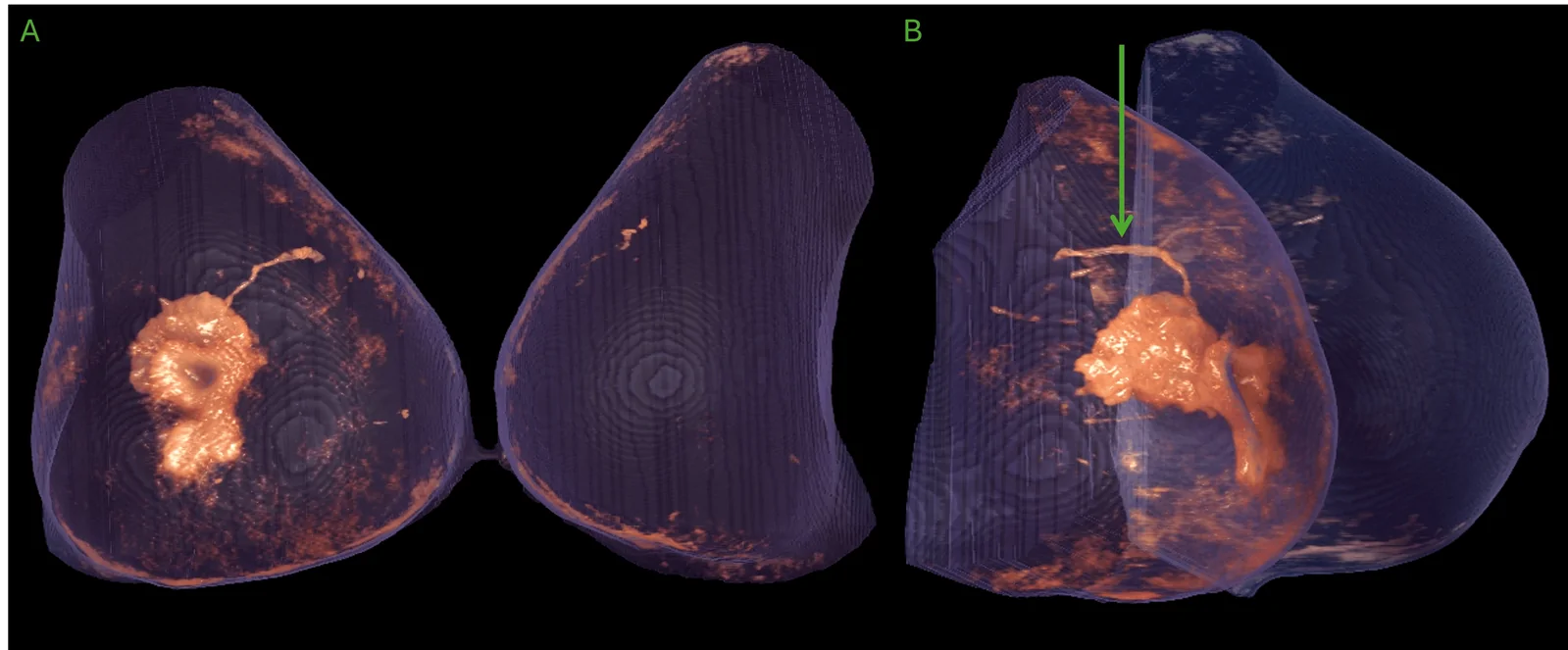
Three-dimensional dedicated breast CT reconstruction demonstrating the right retro-areolar spiculated breast malignancy and its associated vascular supply from the right internal mammary artery. (A) Anterior view. (B) Right lateral view.

Subsequent surgical pathology demonstrated a 49-mm grade 3 invasive breast carcinoma of no special type with associated high-grade ductal carcinoma in situ, giving a total tumor extent of 55 mm. Extensive lymphovascular invasion was present, including dermal lymphovascular invasion. Direct skin invasion with ulceration and three separate dermal satellite tumor deposits were also identified. Three of four right sentinel lymph nodes contained metastatic carcinoma, with extranodal extension present in two involved nodes. The tumor was estrogen receptor positive (90%, 3+), progesterone receptor negative, and HER2 negative (IHC 0). The final pathological stage was pT4b pN1a(sn) according to the eighth edition of the American Joint Committee on Cancer staging system [5]. The difference in progesterone receptor status between the core biopsy and the surgical specimen is consistent with receptor discordance, which may reflect tumor heterogeneity and the sampling differences between the limited core biopsy and the larger surgical specimen [6].

Details of the subsequent multidisciplinary treatment plan and long-term clinical outcome were not available to the authors and therefore could not be reported.

## Discussion

This case demonstrates the potential utility of a dedicated breast spectral CT protocol for improving the visualization and characterization of breast malignancy compared with standard thoracic MonoE40 reconstruction. Although both reconstructions identified the right retro-areolar malignancy, the dedicated breast protocol appeared to provide greater lesion conspicuity on qualitative single radiologist assessment and clearer visualization of enhancement characteristics, associated dermal changes, axillary nodal abnormalities, and tumor vascularity. These additional reconstructions were generated from the same spectral CT acquisition and therefore required no additional radiation exposure, contrast administration, or image acquisition.

Spectral CT provides additional tissue characterization information through monoenergetic reconstruction and iodine mapping, and previous studies suggest that these techniques may improve lesion conspicuity and assessment of enhancement in breast malignancy [3]. In this case, enhancement mapping provided clearer differentiation between the enhancing malignant tissue and surrounding breast parenchyma than the standard MonoE40 reconstruction. Iodine mapping also provided quantitative information, with an average peripheral iodine density of approximately 2.3 mg/mL. Previous breast-specific dual-layer spectral CT research has reported optimal iodine content cutoffs of approximately 0.7-0.9 mg/mL for differentiating malignant from benign breast lesions [7]. Quantitative iodine measurement may therefore provide an additional objective measure for characterizing breast lesion enhancement alongside visual interpretation.

The dedicated protocol also provided additional visualization of the lesion’s irregular spiculated morphology and associated arterial supply from the right internal mammary artery. Three-dimensional vascular reconstruction was performed as an exploratory component of the dedicated breast protocol and provided additional anatomical depiction of the relationship between the tumor and its associated vascularity. Although such vascular mapping may have potential applications in procedural planning, this was not evaluated in the present case, and its clinical significance requires further investigation.

A notable feature of this case was the correlation between the imaging findings and subsequent surgical pathology. Dermal thickening and nipple distortion demonstrated on CT were associated pathologically with direct skin invasion with ulceration, three dermal satellite tumor deposits, and extensive dermal lymphovascular invasion. Similarly, suspicious ipsilateral axillary lymph nodes identified on CT corresponded with metastatic involvement of three of four right sentinel lymph nodes, with extranodal extension present in two involved nodes. Surgical pathology demonstrated a 49-mm grade 3 invasive breast carcinoma of no special type with associated high-grade ductal carcinoma in situ and a total tumor extent of 55 mm, resulting in a pathological stage of pT4b pN1a(sn) [5]. Several abnormalities identified on spectral CT were consistent with the subsequent pathological findings of locally advanced disease. However, conclusions regarding the diagnostic sensitivity, specificity, or staging accuracy of this dedicated breast spectral CT protocol cannot be drawn from a single case.

The maximum lesion dimension measured on CT was approximately 32 mm, whereas surgical pathology demonstrated a 49-mm invasive carcinoma with a total tumor extent of 55 mm. This discrepancy may reflect differences between imaging-based measurement of the dominant visible lesion and pathological assessment of the full tumor extent, including associated in situ and dermal disease. The CT measurement should therefore not be interpreted as representing the complete pathological tumor extent.

Dedicated breast spectral CT reconstruction is not a routinely used primary breast imaging technique in patients with biopsy-confirmed breast cancer. Because breast tissue is frequently included within thoracic CT acquisitions, dedicated spectral reconstruction may nevertheless have a potential future role in opportunistic breast assessment when spectral data are already available. However, this potential role was not established by the present case and requires prospective evaluation. The total dose length product in this case was 330.4 mGy·cm, corresponding to an estimated effective radiation dose of approximately 4.63 mSv. Conventional chest CT has been reported to deliver an effective dose of approximately 8 mSv [4], although dose varies according to patient characteristics, scanner technology, and acquisition parameters. These findings suggest that dedicated breast spectral reconstruction may warrant further evaluation as a potential opportunistic adjunct when spectral data are already available.

This report has several limitations. It describes a single patient and therefore cannot establish diagnostic accuracy or generalizability. Image assessment was not blinded, and no formal quantitative comparison of lesion conspicuity between the standard MonoE40 and dedicated breast reconstructions was performed. Direct comparison with prior mammography, ultrasound, or breast MRI was not possible because the relevant pre-CT breast imaging was not available. Although subsequent surgical pathology provided radiological-pathological correlation for several imaging abnormalities, the study was not designed to determine the accuracy of spectral CT for local tumor or axillary nodal staging. Subsequent oncological treatment and long-term clinical outcome were also unavailable. Prospective studies involving larger patient cohorts, blinded image interpretation, comparison with established breast imaging modalities, and pathological correlation are required to determine the reproducibility, diagnostic performance, and clinical utility of dedicated breast spectral CT reconstruction.

## Conclusions

In this case, a dedicated breast spectral CT protocol incorporating enhancement mapping and three-dimensional reconstruction demonstrated qualitative imaging differences compared with standard MonoE40 reconstruction, including subjectively greater lesion conspicuity and additional depiction of tumor enhancement, dermal abnormalities, suspicious axillary nodes, and vascular supply. Several imaging abnormalities were consistent with subsequent pathological findings, including skin involvement and metastatic sentinel lymph node disease. Because the dedicated reconstructions were generated from spectral data already acquired during the clinically indicated CT examination, they required no additional radiation exposure, contrast administration, or image acquisition. This advantage is specific to examinations in which spectral data have already been acquired and does not necessarily apply to conventional non-spectral CT datasets. This case demonstrates the technical feasibility of dedicated breast spectral reconstruction and qualitative added imaging information in a known biopsy-confirmed breast malignancy undergoing preoperative staging. However, this single case cannot establish clinical benefit, diagnostic superiority, staging benefit, or a defined screening or opportunistic assessment role. Larger prospective comparative studies are required to establish diagnostic performance and clinical utility.
